# Assessing Risk Literacy Skills: Enhancing Healthcare Management among University Students

**DOI:** 10.3390/healthcare12111061

**Published:** 2024-05-23

**Authors:** Diane Dolezel, Ramalingam Shanmugam, Brad Beauvais

**Affiliations:** 1 Health Informatics & Information Management Department, Texas State University, Round Rock, TX 78665, USA; 2 School of Health Administration, Texas State University, San Marcos, TX 78666, USA; shanmugam@txstate.edu (R.S.); bmb230@txstate.edu (B.B.)

**Keywords:** risk literacy, numeracy, Berlin Numeracy Test, college students, decision making

## Abstract

The purpose of this study was to explore the determinants of risk literacy among university students in the United States by utilizing the Berlin Numeracy Test. Risk literacy skills are essential for decision-making and communication of risks, but few studies consider university students. This study aims to evaluate the association of sociodemographic factors with individual risk literacy levels. An observational cross-sectional survey study was used with a convenience sample of 184 undergraduate and graduate university students. Statistical analysis revealed significant differences for demographics at risk for negative outcomes associated with lower risk literacy. For this group of students, the majority had below-average numeracy. These findings can guide healthcare professionals to focus on college-age individuals with low-risk literacy scores to enhance patient understanding, facilitate communication, and promote healthier behaviors.

## 1. Introduction

Risk literacy is a strong multidomain predictor of superior judgment, information processing, and decision-making [1,2,3,4,5]. The ability to understand and apply math concepts, in particular statistics, is the most significant predictor of superior risk literacy abilities [6]. Peters [7] determined that being numerate is essential for decision-making for improved health outcomes. Cokely et al. [1] established that risk literacy (e.g., particularly, statistical numeracy) was strongly correlated with decision-making with information involving statistics, especially statistical probabilities.

Centers for Disease Control [8] defines numeracy as “the ability to access, use, interpret, and communicate mathematical information and ideas, to engage in and manage mathematical demands of a range of situations in adult life” (para. 3). At the lowest level, working with numbers comprises comprehending the number line, understanding time, and correctly using measurements. At a higher level, the emphasis is on statistical numeracy tasks (ratios, proportions, percentages, probability) fundamental to risk analysis such as determining probabilities from frequencies [3].

Prior evidence indicates that many people have low numeracy skills suggesting concern for their risk literacy decision-making skills [5,9,10,11]. Research by the Centers for Disease Control on literacy, numeracy, and problem-solving skills showed that over half of tested U.S. adults had scores in the two lowest scoring levels [8]. The Program for the International Assessment of Adult Competencies mentioned that only 37% of the United States participants aged 16 to 65 had high numeracy scores [12]. In support of this, Lipkus et al. found that 20% of a US general population sample failed to correctly answer questions on risk significance [13].

### 1.1. Importance of Adequate Risk Literacy

Risk literacy skills are essential because the current model of patient-centered healthcare is focused on shared decision-making which involves the patient as part of their care team [14]. With this care model, patients need good risk literacy skills because they will take responsibility for collaborating on treatment decisions and for self-management of their preventative care needs. According to Garcia-Retamero and Cokely (2013) [2], to effectively utilize healthcare information for decision-making, individuals must “understand the risks and benefits of different medical treatments, screenings, and lifestyle choices” (p. 392). Consumers need to comprehend disease risks, medication prescription instructions, diagnostic preparation steps, the risks of different treatment options, and the meaning of graphical presentations of healthcare data [5,15,16]. For example, if they are told the probabilities of side effects for two different drugs (e.g., one in four, etc.) they should be able to determine which drug has the least probability of producing a serious side effect [16].

Low-risk literacy skills are correlated with undesirable patient health outcomes such as poor medical decision-making, an inability to understand disease risks, struggles with medication management, and not understanding diagnostic test results [15,16]. Cavanaugh et al. (2008) [17] determined that older, nonwhite, diabetics with low income and lower education levels had lower numeracy levels which were associated with difficulty managing diabetes due to not recognizing abnormal glucose levels, not managing the amount of carbohydrates in their diet, and mismanagement of their diabetic medications.

Yamashita et al. (2020) analyzed data from the Progamme for the International Assessment of Adult Competencies on sociodemographic factors and utilization of preventative health services for United States adults over the age of 45 years [18]. Their outcome variable was the likelihood of preventative service utilization, and the predictors were numeracy level, health insurance, health literacy levels, age, sex, race/ethnicity, education (college vs. no college), employment status, the number of household members, health insurance, and health literacy levels [18]. Yamashita et al. (2020) determined that higher numeracy levels predicted a greater likelihood of scheduling regular dental visits, but they did not predict higher utilization of other preventative care services (e.g., vision exams, flu immunizations, bone density scans) [19].

Inadequate risk literacy is associated with patient safety. Surgeons need to understand surgical risks and be able to communicate those risks to patients [20]. Surgeons (*n* = 292) from 60 countries were assessed on their risk literacy skills with the BNT, and they were asked to evaluate a case study to compare the risks for postoperative deep vein thrombosis in general anesthesia with a group who had experimental anesthesia. The results of this study indicated several surgeons scored lower on the BNT when compared to the scores for college student participants in similar studies. Moreover, half of the surgeons only had one correct answer (out of four on the BNT) and twenty percent had two correct answers [20].

Petrova et al. (2019) [21] explored the factors that can influence medical residents’ and medical interns’ ability to understand the success of cancer screening results and their ability to make valid patient recommendations on screening. Their study factors were prior beliefs in the effectiveness of screening, understanding of the screening statistics, physicians’ specialties, and their prior statistical education. The results of Petrova et al.’s (2019) study indicated that previous beliefs that cancer screening (in general) was not effective, lower levels of statistical literacy, and lower numeracy skills were significantly associated with lower comprehension of cancer screening results [21]. This study signals a need for more statistical literacy training for physicians during their medical residency and raises concern for patient safety.

### 1.2. Demographics and Risk Literacy

There are a few studies that considered the association of risk literacy and demographics. Durand et al.’s (2020) [22] cross-sectional online survey study utilized several scales to assess the association of graph literacy, numeracy literacy, health literacy, and sociodemographic factors with the understanding of graphic data visualizations for a self-reported Medicaid participant sample in the United States. The Medicaid program provides healthcare to low-income families; thus, one assumption of the study was that participants had a lower socio-economic status [22]. Durand et al. (2020) established that numeracy was associated with gender, education, and health literacy. Moreover, numeracy was positively correlated with graph literacy, but numeracy and health literacy scores were not predictive of a greater understanding of graphs [22].

Friederichs et al.’s (2020) survey study explored the effect of years of clinical experience and the effect of risk literacy levels on the ability to communicate risk related to diseases and treatment choices to patients [23]. Participants were general practitioners and medical students in Germany who took the Berlin Numeracy Test (BNT) and were also assessed on their ability to predict the likelihood that case study patients with positive mammogram test results had breast cancer (i.e., true positive). Demographics were collected for age, gender, and years of clinical experience. Friederichs et al.’s (2020) results showed that BNT scores were positively correlated with the ability to generate the correct true positive mammogram test value for both groups and did not differ significantly by gender [23]. Second, the general practitioners scored higher than medical students on the BNT test, but they did not outperform the medical students when applying those skills to the mammogram screening case scenarios.

Bergner and Filzen [24] determined that age, education, and years of experience since graduating college were associated with BNT scores. Age was negatively associated with BNT scores revealing that younger participants were more likely to have higher BNT scores. Conversely, higher educational attainment was positively associated with higher risk literacy scores. When controlling for age, years of experience were positively correlated with higher BNT scores [25].

### 1.3. College Students and Risk Literacy

There is a need to raise awareness of college students’ risk literacy skill levels because they are an underrepresented population at risk of poor mental and physical health outcomes. At a minimum, attending college exposes students to potential stressors from hectic schedules, moving away from friends and family, changes in sleeping patterns and diets, challenges to getting regular exercise, and stress from academic deadlines. The American Psychological Association [26] reports that during the 2020–2021 academic year, over 60% of all college students surveyed had at least one mental health problem. A Statista [25] survey revealed that 78% of all college students reported they had used tobacco products (e-cigs, cigarettes, cigars, chewing tobacco) in the last 90 days (about 3 months).

Another concern is that college students may no longer be on their parent’s insurance and may need to learn how to self-manage their healthcare for the first time (e.g., make doctor’s appointments, understand health insurance benefits, schedule annual exams, buy medicines, and schedule flu shots). Although student health centers may be available, most charge a fee and there is the consideration of arranging transportation to the healthcare care facility which can be a deterrent from seeking care. Additionally, some college students are single parents or caregivers for family members. These individuals have added responsibilities and numeracy challenges such as understanding pediatrics dosing and assisting elderly relatives with medical billing.

### 1.4. Problem and Purpose

The problem addressed in this study is the gap in the literature on the risk literacy skills of United States college students. Risk literacy skills are essential for decision-making and communication of risks. Traczyk et al. (2022) [27] stated that adequate statistical numeracy is an essential competence because “People with low statistical numeracy have difficulties understanding numerical information” (p. 273). Garcia-Retamero and Cokely asserted that risk literacy skills are required to understand the consequences of medical treatments, lifestyle choices, and missing routine medical screenings [2].

A review of the previous relevant evidence-based literature illuminated the scarcity of studies on the risk literacy skill levels of US adult college students. Research on individual college students’ risk literacy is needed to inform targeted interventions and to remove statistical literacy barriers for college-age consumers. This study aimed to fill the research gap in the body of knowledge on the association of sociodemographic factors with individual risk literacy levels among US adult university students. We assessed the risk literacy levels using the well-validated Berlin Numeracy Test.

### 1.5. Hypotheses

To establish a theoretical background, we consulted the Andersen behavioral model of health service which associates sociodemographic (e.g., age, race, education, gender, income, health insurance) with healthcare service use and health outcomes [28]. Next, we consulted the relevant evidence-based literature to establish which demographics were associated with variations in risk literacy levels.

For the first hypothesis, several studies have found associations with age, race, gender, income, educational level, and health insurance [17,18,22,23]. For example, Yamashita et al. (2020) explored health insurance, age, sex, race/ethnicity, education, and numeracy levels as predictors of healthcare utilization. Cavanaugh et al. (2008) [17] determined that older, nonwhite, diabetics with low income and lower education levels had lower numeracy levels. The related literature considered smoking status [29] and primary language [30] as predictors of health literacy among college students. To our knowledge, there is no literature that considered college or class standing as a factor of risk literacy, so we considered studies in related fields. Thus, for the second and third hypotheses, we conjecture that BNT scores vary with college classification and student classification based on the related health literacy literature [31,32].

Based on our literature review, we conjecture that sociodemographic characteristics are associated with risk literacy skill levels among university students. Therefore, we hypothesize as follows:

**Hypothesis** **1 (H1).** *There will be no association of BNT scores by age, race, gender, insurance, smoking status, income, and language for United States adult college students.*

**Hypothesis** **2 (H2).** *There will be no association of BNT scores by class standing for United States adult college students.*

**Hypothesis** **3 (H3).** *There will be no association of BNT scores by college for United States adult college students.*

In this paper, we first explore relevant research and then the materials and methods used to assess the association between the BNT risk literacy levels and the study factors. Next, the study results are presented. Afterwards, there is a discussion of the research questions and results one by one. The paper closes with a conclusion which contains suggestions for future research.

## 2. Materials and Methods

**Figure 1:**
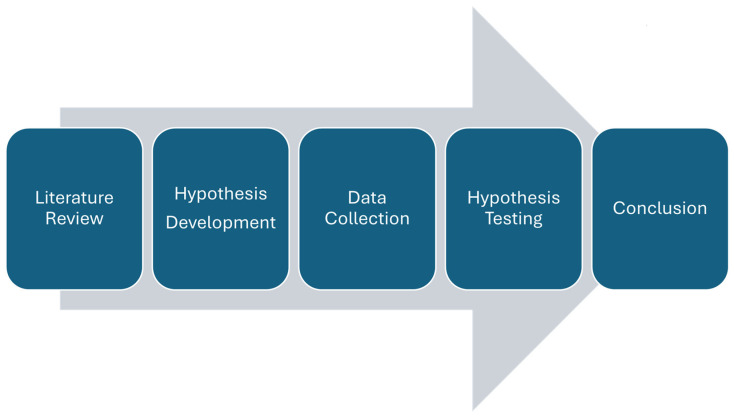
Research process.

A cross-sectional observational correlational survey study evaluated the association of risk literacy levels and sociodemographic factors among adult graduate and undergraduate college students in the United States [1]. Figure 1 presents the research process.

### 2.1. Participants

The study setting was a large state university in the southwestern United States. University students at the study site can complete bachelor’s, master’s, and doctoral levels in a diverse range of programs. The university has a strong commitment to graduate and research and it is classified as a Hispanic-serving institute. The predominant university population demographic is White and non-Hispanic (43.4%). Forty percent of the university students are female, and most students are 18–24 years old.

With IRB approval, a listing (*n* = 4000) of graduate and undergraduate college students at the study site was selected from an administrative system and the students were recruited by email to participate in the survey in the Summer of 2023. The study participants were a convenience sample who indicated they wanted to be in the study by responding to these email invitations. Respondents could take the survey based on two criteria: they designated themselves as 18 years or older (within the survey) and indicated a willingness to participate by selecting a survey button on the first page.

### 2.2. Research Tools

The BNT online survey was self-administered with Qualtrics 2024 survey software. Survey data was exported from Qualtrics and imported into a Microsoft Excel 2024 spreadsheet. The Excel data were analyzed with R statistical software 2023 [33] and with IBM Corp. SPSS Statistics Version 28 [34].

### 2.3. Data Collection and Processing

Data were collected with a Qualtrics online survey at the study site [35]. The study survey, designed by the researchers, contained all the BNT questions in addition to self-reported general demographics (age, race, gender, income, primary language), health demographics (i.e., health insurance, and smoking status), class standing, and college. There were 184 valid responses analyzed, providing a response rate of 4.6%.

### 2.4. Dependent Variable

The dependent variable in our study was the total score on Cokely et al.’s (2012) Berlin Numeracy Test [1]. The BNT has four multiple-choice case study questions, which each have four response choices, which assess their numeracy knowledge on probabilities [36]. Each correct question is worth 1 point, and incorrect questions were assigned 0 points, resulting in a total possible BNT score of 0–4 points. To illustrate, a student who answered two questions correctly would have a BNT score of two. A score of zero indicates no apparent numeracy abilities and a score of four indicates the maximum numeracy ability, there was no other meaning assigned by the BNT developers. Cokely et al. reported an average BNT score of 1.61 points (*SD* = 1.21) [1].

The Berlin Numeracy Test is a validated and reliable instrument for assessing statistical risk literacy (the ability to use statistical test results for risk interpretation). Cokely et al. [1] validated the BNT for use with highly educated (i.e., some college) populations over 18 years of age in 15 countries including Germany, England, Norway, France, and Switzerland. Friederichs et al. [36] assessed risk literacy among German medical students on their ability to understand and communicate risk probabilities to patients. A related study in Germany explored statistical literacy levels for general practitioners compared to third-year medical students utilizing the BNT [23].

The BNT has double the predictive power of related numeracy and cognitive tests [6]. Evidence-based studies utilizing the BNT have demonstrated the discriminant, convergent, and criterion validity of this instrument [6]. A strength of the BNT is that it is intended to estimate the variance in statistical risk literacy among educated individuals (e.g., college students, professionals in business, medicine, or the legal fields). A limitation of the BNT is that it is not suitable for assessing individuals with high school or lower educational levels. Another limitation of the BNT is that it provides a broad assessment of individual risk literacy levels, but it does not offer any details on reasoning deficiencies in specific areas of statistical risk literacy [1].

### 2.5. Independent Variables

Previous empirical research guided the selection of the independent variables [22,23,30,37,38]. The independent variables were categorical age, gender, health insurance, smoking, race, household income in US dollars, primary language spoken in the household class standing, and the college the student was enrolled in for their major. Age was coded as a categorical variable (0 = ‘18–21’, 1 = ‘22–50’). Gender, health insurance, and smoking were binary variables (0= ‘No’, 1 = ‘Yes’). Guided by Hoover et al. [29], smoking was assessed on whether the individual reported they had smoked 100 or more cigarettes in their lifetime.

Race was initially categorized as White, Black, African American, Hispanic, Asian, Native Hawaiian or Pacific Islander, American Indian, and Other. However, due to low frequencies, it was collapsed to (1 = ‘White’, 2 = ‘Hispanic’, 3 = ‘Other’). Household income in US dollars was stratified as ‘Less than USD 50,000’ and ‘More than USD 50,000’.

**Figure 2:**
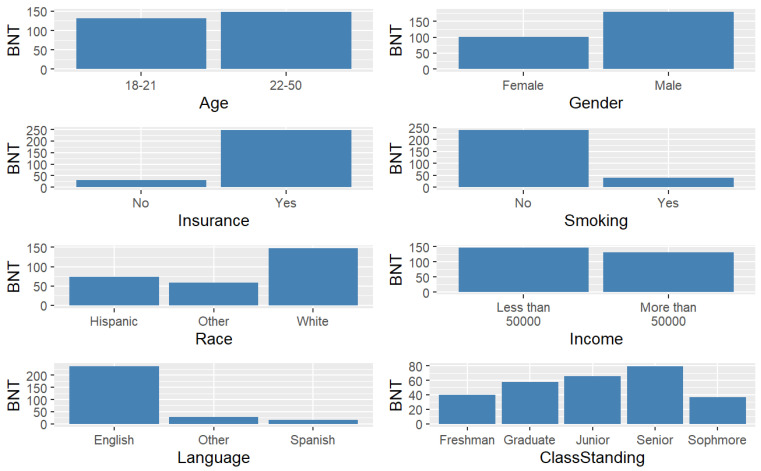
Frequencies for independent variables.

**Figure 3:**
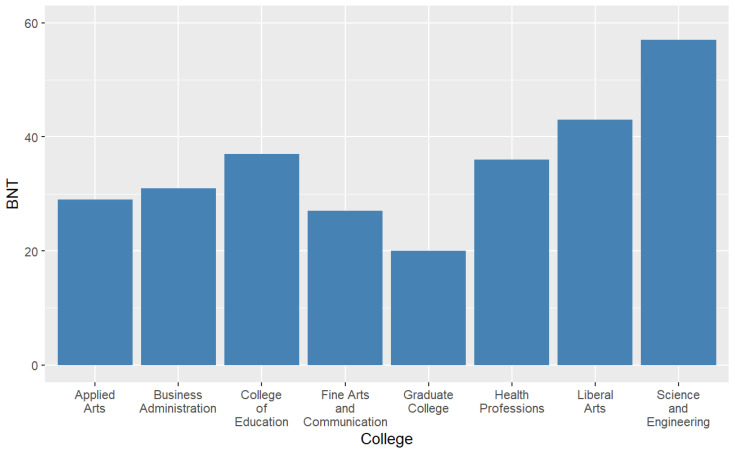
Frequencies for college.

The primary language was coded as ‘English’, ‘Spanish’, and ‘Other’. Class standing was grouped as freshman, sophomore, junior, senior, and graduate. The response choices for college were ‘Applied Arts’, ‘Business Administration’, ‘Education’, ‘Fine Arts and Communication’, ‘Health Professions’, ‘Liberal Arts’, ‘Science and Engineering’, and ‘Graduate College’. Figure 2 and Figure 3 display the frequencies for the independent variables.

### 2.6. Data Analysis

Data were analyzed with IBM Corp. SPSS Statistics Version 28 [34] and with R statistical software 2023 [33]. There was no missing data for the dataset (*n* = 184). The BNT test was scored with Qualtrics 2024 software. All levels of the BNT Score (zero to four) were represented in the independent variables. Relationships between the BNT scores and the independent variables were analyzed at the alpha significance level of 0.05 (*p* ≤ 0.05).

First, sample descriptives were generated to describe the sample characteristics with frequencies and percentages. Second, BNT score descriptives and graphs provided a picture of the BNT score distribution. Third, risk literacy score statistics (mean, SD, minimum, and maximum) were reported for each independent variable for all categorical levels. Fourth, ANOVA tests were used to examine the association between the dependent and the independent variables age, race, gender, insurance, smoking, income, or language. The one-way ANOVA test is the appropriate statistical test for comparing two or more means of a numeric dependent variable (the BNT score) on the categorical independent variable groups [39].

Fifth, ANOVA tests and linear regression were used for association tests between BNT score, class standing, and college. Sixth, several partial correlations were calculated to explore the relationships between the BNT score and age while controlling for race, gender, insurance, smoking, income, language, class standing, and college, respectively. Seventh, factor analysis was conducted to explore the proximity of the independent variables to the BNT score.

## 3. Results

### 3.1. Sample Descriptives

Table 1 displays the sample descriptives. There were 184 respondents, 69% males and 31% females. Their ages ranged from 18 to 49 years (*M* = 22.63, *SD* = 5.79), and the majority (52.7%) were 18–21 years of age. Half were White (50.0%) followed by Hispanic or Latino (29.9%). The predominant language was English (83.7%), and the majority had health insurance (87.0%). Less than 13% reported smoking over 100 cigarettes in their life. Approximately 44.6% had household incomes over USD 50,000 annually. About half of the respondents were juniors (21.7%) or seniors (28.8%). Most students were in the College of Liberal Arts (18.5%) or the College of Science and Engineering (17.4%).

### 3.2. Dependent Variable Descriptives

**Figure 4:**
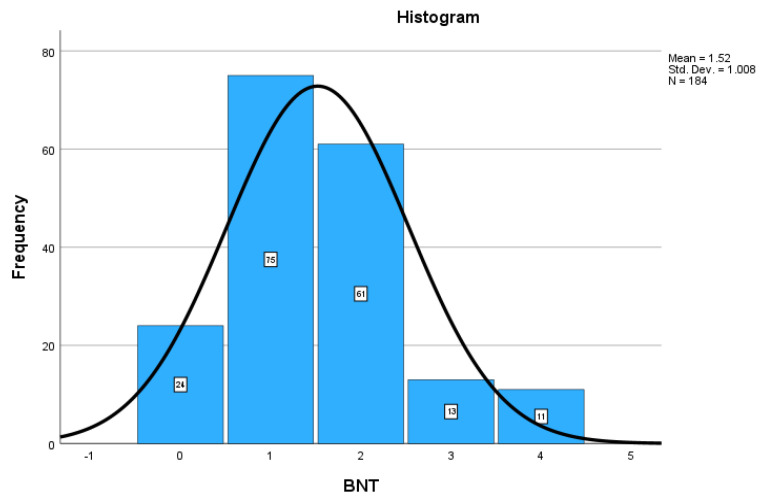
Distributions of the BNT Score.

Figure 4 presents the approximately normal distribution of the dependent variables and the total score on the Berlin Numeracy Test for the 184 respondents. The BNT scores ranged from 0 to 4, with a mean of 1.52, and a standard deviation of 1.01. The median and mode were both 1.00. Most respondents (53.8%) had below-average risk literacy when compared to those with above-average numeracy (46.3%). Specifically, twenty-four respondents (13%) had zero scores, indicating no risk literacy ability (i.e., all four BNT question responses were incorrect). Seventy-five respondents (40.8%) had one question correct, and sixty-one (33.2%) had two questions correct on the Berlin Numeracy Test. Thirteen respondents (7.1%) had a score of three on the BNT. A perfect score of four (i.e., all four BNT question responses were correct) was earned by eleven respondents (6.0%), indicating the highest level of risk literacy.

Regarding the normality of the BNT score distribution, in a normal distribution the mean, median, and mode are all the same. For our sample distribution, the median and mode were both 1.00 but the mean was 1.52. However, the standard error of the mean (*SE* = 0.074) was close to zero providing evidence for BNT score normality. Additionally, the skewness was 0.652, and Hair et al. [34] stated that skewness values between −1 and +1 are excellent. The kurtosis value was 0.312 which is <3 implying the BNT score data has fewer extreme values than a normal distribution. Thus, we consider the BNT distribution an approximately normal distribution, which was confirmed by a visual examination of a P-P plot.

### 3.3. BNT Scores by Independent Variable

**Figure 5:**
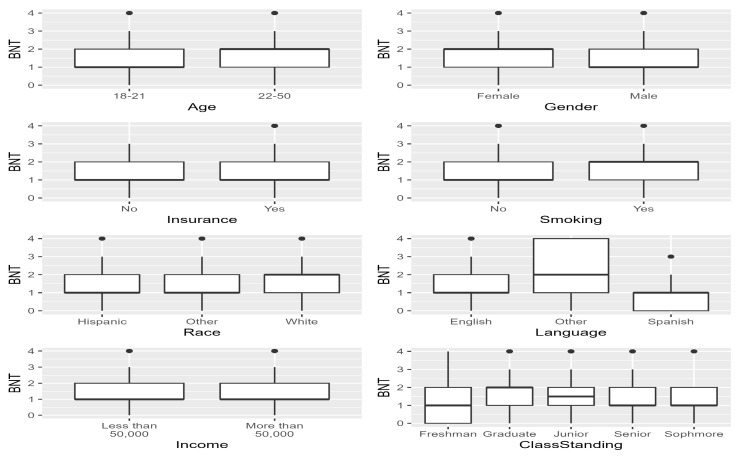
BNT scores for the independent variables (age, gender, insurance, smoking, insurance, race, income, language).

**Figure 6:**
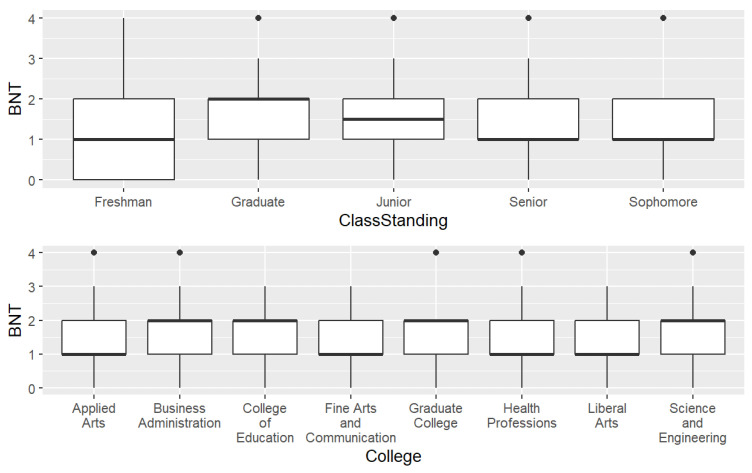
BNT Scores for the independent variables college and classification.

Figure 5 and Figure 6 display the BNT Scores by independent variable with grouped boxplots where black dots indicate potential outliers. First, we examined the grouped box plots for the BNT score and each independent variable. Overall, the highest BNT score (4) was present in most of the independent variables for all levels, except Insurance = ‘No’, freshman class standing, and some colleges. The median BNT score was higher for ages 22–50 when compared to the 18–21 group.

Females had higher median scores than males. The median for Insurance versus no insurance was the same for all levels. Smokers had higher median scores when compared to nonsmokers. Whites had median scores of two, the highest median score among the race levels. Considering primary language, the ‘Other’ group had the largest variance in scores and the highest median score. The median for both income levels was the same. Graduates had a median score of two, the highest for this group, and first-year students had the highest zero scores.

Table 2 presents the BNT scores statistics for each independent variable. First, we noted the highest and lowest average scores for all the independent variables. The highest average BNT score for any categorical independent variable level occurred for the primary language variable group Other (*M* = 2.08, *SD* = 1.44). The lowest average score for any level of any independent variable occurred for the primary language group of Spanish (*M* = 1.00, *SD* = 0.87).

Next, we considered each independent variable independently. The highest average BNT score by age was for the 22–50-year age group (1.70). Females (1.77) had higher scores than males. Non-smokers (1.74) had higher scores than smokers. As expected, individuals with health insurance had higher scores (1.55) when compared to those without health insurance. The race of White (1.61) has the highest score among the races. Income over USD 50,000 per household (1.61) had higher scores than those with incomes less than or equal to USD 50,000 per household.

The primary language other (2.08) classification had the highest score among their categorical variable levels in those variables. As estimated, graduate students had the highest scores (1.76) among all the class standings. And among the different university colleges, the graduate college students had the highest scores (1.82).

### 3.4. Risk Literacy Levels by Age, Race, Gender, Insurance, Smoking, Income, or Language

First, one-way analysis of variance (ANOVA) tests were conducted to examine the relationships of age, race, gender, insurance, smoking, income, or language on risk literacy scores, as measured by the BNT, at the significance level of 0.05. Second, a post hoc Tukey HSD test was conducted to explore any significant results. Overall, age, gender, and primary language were the only variables found to be significant. Conversely, the correlations between race (*p* = 0.30), insurance (*p* = 0.33), smoking (*p* = 0.27), and income (*p* = 0.29) were insignificant.

The independent variable age was coded as 18–21 (*n* = 97) and 22–50 (*n* = 87) years. There was a statistically significant difference between age groups as determined by a one-way ANOVA at (*F* (1, 182) = 5.35, *p* = 0.022). The post hoc test indicated significant pairwise differences in BNT scores between the age groups, the 22–50 years age group (*M* = 1.70, *SD* = 1.02) showed, on average, a higher BNT score (*Mdiff* = 0.34) when compared to the 18–21-year-old group (*M* = 1.36, *SD* = 0.96) This indicates that respondents in the 22–50 years age group had higher BNT scores on average when compared to the 18–21 years age group.

Gender was classified as female (*n* = 57) and male (*n* = 127). There was a statistically significant difference between gender groups as determined by one-way ANOVA at (*F* (1, 182) = 5.21, *p* = 0.024). The post hoc HSD test indicated significant pairwise differences in BNT scores between the genders, males (*M* = 1.41, *SD* = 0.96) had on average lower BNT scores (*Mdiff* = −0.36). when compared to the females (*M* = 1.77, *SD* = 1.07).

Primary language was classified as English (*n* = 154), Spanish (*n* = 17), and other (*n* = 13). The correlation between primary language spoken at home is very significant at (*F* (2, 181) = 4.42, *p* = 0.013). The follow-up test indicated significant pairwise differences in BNT scores between Spanish speakers and the other language speaker group. Spanish speakers (*M* = 1.00, *SD* = 0.87) had on average lower BNT scores (*Mdiff* = −1.08). when compared to the other group (*M* = 2.08, *SD* = 1.44). The English group (*M* = 1.53, *SD* = 0.98) did not differ significantly from the Spanish (*p* = 0.09) or other groups (*p* = 0.14) on BNT scores. In summary, for this sample, the other group had significantly higher BNT scores when compared to the English and Spanish groups.

### 3.5. Risk Literacy Levels by Class Standing and College

The one-way ANOVA test between class standing and BNT score was insignificant (*p* = 0.316). This is surprising because age was positively associated with BNT score, and older students would be predominant among the students classified as graduate (i.e., attending graduate college). To further explore the relationship between classification and BNT score, a linear regression was conducted with BNT score as the dependent variable, classification as the predictor, and a reference level of ‘Freshman’. Results of the linear regression indicated that the overall regression model was insignificant (*p* = 0.32), and the only significant variable level was the graduate classification (*p* = 0.065 < 0.1).

Regarding college, we expected that majors attending science and engineering-oriented, or graduate colleges would be associated with a higher score on the BNT risk literacy test. Surprisingly, college was not a significant predictor of BNT score (*p* = 0.323).

### 3.6. Partial Correlations

Partial correlations were calculated to explore the relationships between the BNT score and age while controlling for race, gender, insurance, smoking, income, language, class standing, and college. For example, if age is the principal predictor of BNT score we expect the partial correlations of age and BNT score to be insignificant or there will be a small effect on the strength of the zero-order correlations in comparison to the partial correlation coefficients.

A partial correlation was conducted for BNT score and age, holding college constant. The zero-order correlation between BNT score and age was *significant* (*r* (*184*) = 0.198, *p* = 0.007). The correlation between BNT score and college was *insignificant* (*p* = 0.995). The correlation between college classification and age was insignificant (*p* = 0.060). The partial correlation between BNT score and age was *significant* (*p* = 0.007, *r* (*184*) = 0.198) when holding college constant but there was no change in the strength of the zero-order correlations indicating that controlling for college had little effect on the relationships between BNT score and age.

A partial correlation was conducted for BNT score and age, holding class standing constant. The zero-order correlation between BNT score and age was *significant* (*r* (*184*) = 0.198, *p* = 0.007). The correlation between the BNT score and class was *insignificant* (*p* = 0.078). The correlation between class and age was very significant (*p* < 0.001). The partial correlation between BNT score and age was *significant* (*p* = 0.038, *r* (*184*) = 0.154) when holding college constant and there was a small decrease in the strength of the zero-order correlations (from 0.198 to 0.154) indicating that controlling for the class had little effect on the relationships between BNT score and age.

A partial correlation was conducted for BNT score and age, holding gender constant. The zero-order correlation between BNT score and age was *significant* (*r* (*184*) = 0.198, *p* = 0.007,). The correlation between BNT score and gender was *significant* (*p* = 0.024). The correlation between gender and age was insignificant (*p* = 0.061). The partial correlation between BNT score and age was *significant* (*p* = 0.015, *r* (*184*) = 0.179) when holding gender constant and there was a small decrease in the strength of the zero-order correlations (from 0.198 to 0.179) indicating that controlling for gender had little effect on the relationships between BNT score and age.

Five additional individual partial correlations were computed for each of these control variables: race, insurance, smoking, income, and primary language. For each partial correlation, the zero-order correlations of BNT score and age were significant, but a comparison of the partial correlations to the zero-order correlations demonstrated little change from the strength of the zero-order correlations when holding the corresponding control variable constant. This suggests that controlling those variables had little effect on the strength of the relationship between BNT score and age.

### 3.7. Factor Analysis

To explore the proximity of the variables to the BNT score, we did a factor analysis with principal component analysis (PCA) in SPSS. The Kaiser–Meyer–Olkin value of 0.554 indicated sampling adequacy, and a statistically significant Bartlett’s test of sphericity < 0.001 supported factoring the correlation matrix [40].

PCA revealed four components with eigenvalues greater than one which explained 55.4% of the total variance. Any factors with loadings below 0.3 or with multiple loadings above 0.3 were removed from the analysis. Thus, insurance and class standing were removed from the analysis because they were loaded on two constructs with loadings greater than 0.3. Based on the Scree plot bend, three factors were extracted. The factors retained were component 1 (income, smoking, college), component 2 (race and primary language), and component 3 (gender).

**Figure 7:**
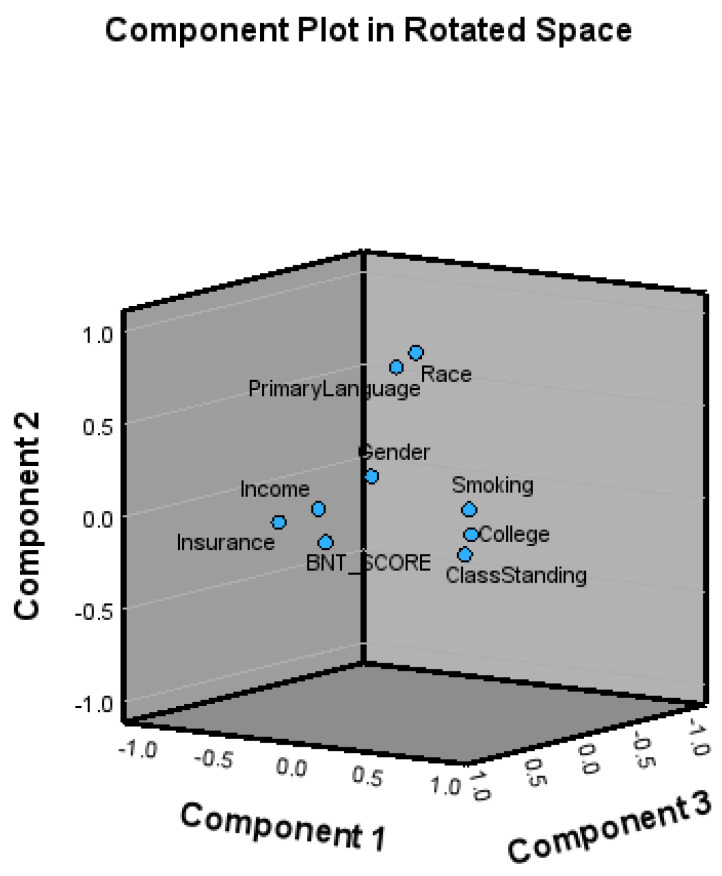
Three-dimensional proximity of variables to BNT score.

Figure 7 presents a three-dimensional plot of the proximity of the variables, based on 47.15% of the total variations. There is a closer proximity for income and insurance to the primary variable which is the BNT score. Gender is close to the BNT score. Race and primary language are close to each other. Class standing is close to college as expected.

## 4. Discussion

This study explored the risk literacy levels of undergraduate and graduate college students in the United States with the Berlin Numeracy Test. To our knowledge, this study is unique because it is the first study to use the BNT to assess numeracy risk skills for individual adult college students in the United States. Our study had significantly more students with BNT scores (53.8%) below the mean as compared to those with BNT scores above the mean (46.3%), indicating that respondents in this sample did not have adequate risk literacy skills. The average BNT score for our study was *M* = 1.52 (*SD* = 1.008) which is lower than Cokely et al.’s study average score of 1.6 points (*SD* = 1.21) [1].

### 4.1. Principal Findings

First, the results support rejecting the first null hypothesis because there were several significant sociodemographic factors associated with the BNT test scores. Second, the results did not support rejecting this hypothesis as the correlation between class standing and BNT score is insignificant. Third, the results did not support rejecting this hypothesis which implies the relationship between college and BNT score is insignificant. Fourth, the partial correlations used to explore the relationships between the BNT score and age suggest that controlling for the other study variables had little effect on the correlation between the BNT score and the age of the respondent in years. Fifth, the factor analysis revealed that three components explained approximately 55.4% of the total variance in the BNT score.

### 4.2. Discussion of Results by Hypotheses

For this study, we examined risk literacy for individual college students. There are no directly comparable studies conducted on college students in the US. Most research in this area considered numeracy scores at the group (not individual) level for medical students [23], physicians [21], C-suite risk numeracy [24], or college students [1] outside the United States. There were only a few (non-US studies) that considered respondent demographics as predictors of BNT scores and they were not for the general college population [22,23,36].

Our results revealed that age, gender, and primary language are significantly associated with higher risk literacy levels as measured by the BNT. Conversely, race, insurance, smoking, and income were not significant predictors of BNT scores in this sample. The demographic subgroups with the highest BNT test scores were females, individuals in the 22–50-year age group, and those in the other language group. Our study found that female college students had higher estimated median BNT scores than males. In contrast, Friederichs et al. [36] assessed German medical students with the BNT to establish that males had significantly higher numeracy scores than females. Durant et al. also found that females had higher odds of having lower numeracy scores among a Medicaid-eligible population [22]. However, other studies found no significant differences in BNT scores by gender for General Practitioner physicians as compared to the BNT scores for medical students [23]. This diversity of results on gender indicates a need for further exploration of the association between gender and BNT scores.

Regarding the difference between these three prior studies and our study, there is a disparity in sample demographics and study settings (i.e., German medical students (51% female), German practitioners (60% female), and Medicaid eligible group (82% female) that makes it challenging to compare their results to our study which surveyed United States college students from various academic disciplines within a public university. For example, our study was 39% female and the prior studies had significantly more female representation. Two studies were conducted in Germany (with medical students and with doctors), and one study assessed lower socio-economic groups (i.e., Medicaid eligible), but we sampled United States college students from various colleges with over 44% having household incomes of more than USD 50,000.

Interpreting age, our study determined that older students had higher BNT scores. In contrast, Bergner and Filzen [24] examined C-suite risk numeracy among business executives and professionals with BNT and found that age was negatively associated with numeracy indicating their older respondents had lower BNT scores. However, the Bergner and Filzen [24] study had a much higher average age ($\bar{x}$ = 54) than this study ($\bar{x}$ = 22.63), and one limitation was their smaller sample size (*n* = 77).

The second hypothesis was supported because our results suggested the relationship between risk literacy levels and class standing was insignificant. The third hypothesis was supported because the study results indicated no significant association between risk literacy levels and class standing. In contrast, results from Rababah et al.’s (2019) study found that college and class standing were significant predictors of health literacy [30]. Their results showed that students in health-related majors had significantly higher health literacy scores when compared to those in non-health-related majors, and students with higher class standing scored higher on the health literacy test [30]. However, Rababah et al.’s (2019) study had a larger sample (*n* = 520) than our study and their levels for field of study only considered health-related versus others (engineering, sciences). In congruence, the National Assessment of Adult Literacy found a strong correlation between a higher level of education attainment and average health literacy college, but their relevant education level classifications (some college, associate’s, bachelor’s, graduate) did not provide the class standing (freshman, sophomore, junior, senior) [32].

For our study, we expected that students attending health and technical colleges (e.g., Health Professions, Engineering, Business Administration) would have higher risk literacy scores but this was not the case. Students enrolled in graduate programs (*n* = 11) had the highest BNT scores, followed by Science and Engineering (*n* = 32) and Business Administration (*n* = 18) students. Overall, the range of scores for class standing was 1.29 (freshman) to 1.76 (graduate students). It is possible that a larger sample with a similar number of students selected from each college and each class standing could have provided different results.

### 4.3. Practical Implications

Risk literacy is an important skill for professional and everyday decision-making. Despite the growing concern that the US risk literacy levels are low, there is limited research on this topic for college students. Our findings suggest that age, gender, and primary language are associated with numeracy levels, which indicates a need for interventions to reduce this inequality. Younger college students may need more support to master mathematical and statistical risk skills. Males may need study materials to help them with risk literacy concepts. Interventions to improve risk literacy could include online statistical training tutorials and testing, gamification of statistical learning with interactive study tools, and educational applications to teach statistics. Clinical personnel may wish to assess the patient’s risk literacy skills with the BNT.

### 4.4. Limitations

This study has some limitations. First, the sample for this study was drawn from a single public university. Additional samples drawn from a more diverse set of collegiate settings across geographic regions might produce different results from our study findings. Second, most respondents in our study were male, and most reported English as their primary language. The BNT is written in English, so this could lead to higher scores in comparison to a more culturally diverse group. Third, a larger sample size also could have revealed new relationships between the independent variables and the BNT scores. Further, a larger sample might have produced an increased effect size for other predictors in the study and a stratified sample with equal gender groups may have produced more meaningful results. Fourth, the survey responses are self-reported and may reflect the respondents’ biases. Fifth, this was a cross-sectional study (i.e., point in time) which may not represent the overall association of the studied demographics and the BNT scores over time, for that a longitudinal study is needed. External validity could have been assessed more fully if there were results available for similar populations. Finally, this non-experimental study cannot determine cause and effect.

## 5. Conclusions

This study provided empirical evidence on the demographic factors among college-age students that may present barriers to their ability to access, use, and interpret numeric information effectively to manage their healthcare and their daily needs by utilizing the Berlin Numeracy Test. The strength of this study is the use of the BNT in the United States. To our knowledge, it is the only BNT study conducted in the US and one of only a few studies assessing a diverse group of US college students with the BNT. We found that age, gender, and primary language were significant predictors of risk literacy level. Conversely, race, insurance, smoking, and income were not significant predictors of risk literacy for this sample. Our results presented significant differences for demographics at risk for negative outcomes associated with lower risk literacy which provide valuable information for designing interventions to improve health outcomes. These results may guide college educators, curriculum developers, and clinical personnel who aim to improve risk literacy skills.

### Future Research

As we noted, the BNT test results provide a total score that can be used to evaluate the individual’s general risk literacy skill level. However, it does not provide details on the logic deficiencies in a specific area of risk literacy which creates a gap to be filled by future research. Another, more ambitious undertaking for future studies would be establishing the associations between risk literacy and risk decision-making. For this endeavor, consideration might be given to interventional studies with BNT testing before and after the intervention, and to BNT testing followed by the completion of practical risk-based case scenarios to assess the areas of risk literacy deficits. Interventions could include talk-back and teach-back exercises to assess the individual’s ability to apply risk literacy skills. Multisite US college studies would facilitate generalizing the BNT to US college populations. Future research should explore larger US college student populations, surveys at multiple US colleges, and interventional studies to improve risk literacy. College courses should assess students with the BNT, they could then provide targeted content on statistical numeracy.

## Figures and Tables

**Table 1:** Sample demographics characteristics (*n* = 184).

Characteristics	*n*	*%*
Age (years) *M* = 22.63, *SD* = 5.79 (reported in years) *M* = 22.63		
18–21	97	52.7
22–50	87	47.3
Gender		
Male	127	69.0
Female	57	31.0
Smoking Status		
Yes	23	12.5
No	161	87.5
Health Insurance		
Yes	160	87.0
No	24	13.0
Race		
White	92	50.0
Black or African American	13	7.1
Hispanic or Latino	55	29.9
Asian	17	9.2
American Indian or Alaskan Native	2	1.1
Other	5	2.7
Income (US Dollars)		
Less than 10,000	27	14.7
10,000–25,000	26	14.1
26,000–50,000	32	17.4
More than 50,000	82	44.6
Do not wish to answer	17	9.2
Primary Language		
English	154	83.7
Spanish	17	9.2
Other	13	7.1
Class Standing		
Freshman	31	16.8
Sophomore	27	14.7
Junior	40	21.7
Senior	53	28.8
Graduate student	33	17.9
College		
College of Applied Arts	17	9.2
College of Business Administration	18	9.8
College of Education	25	13.6
College of Fine Arts and Communication	20	10.9
College of Health Professions	27	14.7
College of Liberal Arts	34	18.5
College of Science and Engineering	32	17.4
Graduate College	11	6.0

**Table 2:** BNT score statistics by independent variables (*n* = 184).

Independent Variables	N	Mean (SD)	Min.	Max.
Age (years) (reported in years) *M* = 22.63				
18–21	97	1.36 (0.96)	0	4
22–50	87	1.70 (1.02)	0	4
Gender				
Male	127	1.41 (0.96)	0	4
Female	57	1.77 (1.07)	0	4
Smoking Status				
Yes	23	1.49 (1.01)	0	4
No	161	1.74 (0.96)	0	4
Health Insurance				
Yes	160	1.33 (0.87))	0	3
No	24	1.55 (1.02)	0	4
Race				
White	92	1.61 (0.99)	0	4
Hispanic or Latino	55	1.57 (1.12)	0	4
Other	37	1.35 (0.97)	0	4
Income (US Dollars)				
Less or Equal 50,000	102	1.45 (0.98)	0	3
Greater than 50,000	82	1.61 (1.04)	0	4
Primary Language				
English	154	1.53 (0.98)	0	4
Spanish	17	1.00 (0.87)	0	3
Other	13	2.08 (1.44)	0	4
Class Standing				
Freshman	31	1.29 (1.13)	0	4
Sophomore	27	1.37 (0.79)	0	4
Junior	40	1.65 (1.08)	0	4
Senior	53	1.49 (0.93)	0	4
Graduate student	33	1.76 (1.06)	0	4
College				
College of Applied Arts	17	1.71 (1.36)	0	4
College of Business Administration	18	1.72 (0.96)	0	4
College of Education	25	1.48 (0.82)	0	4
College of Fine Arts and Communication	20	1.35 (0.81)	0	4
College of Health Professions	27	1.33 (1.00)	0	3
College of Liberal Arts	34	1.26 (0.83)	0	3
College of Science and Engineering	32	1.78 (1.01)	0	4
Graduate College	11	1.82 (1.33)	0	3

## Data Availability

The original contributions presented in the study are included in the article, further inquiries can be directed to the corresponding author.
